# DANCER: Study protocol of a prospective, non-randomized controlled trial for crossed limb versus standard limb configuration in endovascular abdominal aortic aneurysm repair

**DOI:** 10.3389/fcvm.2022.1046200

**Published:** 2022-11-09

**Authors:** Yinzhi Shen, Jiarong Wang, Jichun Zhao, Ding Yuan, Tiehao Wang, Bin Huang

**Affiliations:** ^1^West China School of Medicine, Sichuan University, Chengdu, China; ^2^Department of Vascular Surgery, West China Hospital, Sichuan University, Chengdu, China

**Keywords:** non-randomized controlled trial, crossed limb, standard limb, EVAR, abdominal aortic aneurysm

## Abstract

**Background:**

Hostile anatomy, especially severely angulated neck and tortuous iliac arteries, has always been a conundrum in endovascular aneurysm repair (EVAR). Crossed limb (CL) graft, also called the “ballerina technique,” has been utilized to address this problem by facilitating gate cannulation. In terms of short and long-term outcomes, correlated studies have made inconsistent conclusions and this issue remains controversial. Based on a previous cohort study conducted in our center, we aim to prospectively compare the safety and efficacy between CL and standard limb (SL) configuration in patients receiving EVAR.

**Methods:**

This is a prospective, single-center, non-randomized controlled trial. A total of 275 patients who meet the inclusion criteria will be enrolled and allocated with a 4:11 ratio of CL to SL, which is based on results of our previous study. All patients will receive same perioperative management and postoperative medications. All EVAR procedures will be performed under standard protocol, utilizing Endurant II or IIs Stent Graft. The configuration of the graft stent will be decided by surgeons and confirmed by final angiography. The primary outcome is 3-year freedom from major adverse limb-graft events (MALEs). Endpoints will be assessed at the following time points: 1, 6, 12, 24, and 36 months.

**Discussion:**

To our best knowledge, this crosseD vs. stANdard Configuration in Endovascular Repair (DANCER) trial is the first non-randomized controlled trial to compare these two graft configurations in EVAR. The main aim is to compare the MALEs between two groups at 3 years postoperatively. This trial will hopefully provide high-level evidence for employing CL in EVAR.

**Clinical trial registration:**

[www.chictr.org.cn], identifier [ChiCTR2100053055].

## Introduction

Abdominal aortic aneurysm (AAA) is a pathological, localized dilation of abdominal aorta (1), characterized by decreased smooth muscle cells, extracellular matrix breakdown, inflammatory cell infiltration and endothelial dysfunction (2). The prevalence of AAA increases with age, estimated to be 1.3–1.7% in 65-year-old men (3, 4). Many patients with AAA are asymptomatic and mostly diagnosed after incidental imaging or, in the worst case, rupture (1). With mortality up to 85%, AAA rupture is an important death cause in adults, which renders AAA one of the most important diseases in vascular surgery (5). Both the Society for Vascular Surgery and the European Society for Vascular Surgery have recommended endovascular aneurysm repair (EVAR) as the preferred treatment modality for AAA in certain patients (6, 7). Compared with traditional open surgical repair, EVAR has the advantage of minimal invasiveness and shorter procedure time (8). Nevertheless, EVAR performed in patients with hostile anatomy could be technically challenging and more likely to induce postoperative complications. A large number of studies concluded that hostile proximal neck is associated with a higher rate of type I endoleak, secondary procedure and mortality in the short and long term after the procedure (9–11). Similarly, distal aortoiliac tortuosity has been revealed to be related with higher graft-related complications (12–14). To resolve this problem, great innovations have been made in the past few decades and one of them, crossed limb (CL), was raised in 2002 for patients with severely angulated neck. This ballet-dancer-like configuration could facilitate gate cannulation and theoretically avoid graft disconnection and endoleaks (15). By now, this technology has been widely applied in clinical practice.

For efficacy of the CL in practice, however, related studies have drawn inconsistent conclusions, which makes the true effect of CL doubtful. By analysis of computed fluid dynamics, CL showed a tendency to prevent stent thrombosis, accompanied by tolerance for higher wall shear stress and helicity characteristics (16, 17). On the contrary, long-term fatigue and displacement implications were revealed by the same studies and another one demonstrated that it was the angle of the aneurysm neck other than the configuration that affected hemodynamic index (18). Similar controversial outcomes were observed in clinical trials. Two retrospective cohort trials showed that CL group had a longer procedural time and more type II endoleak compared with standard limb (SL) groups; however, technical success rate, postoperative complications, reintervention and overall survival were comparable (19, 20). This result was mirrored in the latest meta-analysis with no significant difference found in perioperative mortality, endoleak or limb occlusion. This study concluded that CL did not confer inferior clinical outcomes compared to SL in the medium term (21).

To further explore this issue, we conducted a cohort study in 2021, which suggested that no significant evidence was found to favor either configuration in terms of adverse limb events, endoleak, reintervention or overall survival (22). A trend toward a lower risk of type IB endoleak was observed in the CL group after stratification by large aneurysm sac or tortuous iliac artery. On the other hand, CL incurred a higher risk of reintervention and adverse limb events in patients with angulated aneurysmal neck. As commented by editors, our previous study showed interesting results and had the advantage of significant enrolled patients and sufficient time of follow up. However, it was subject to its retrospective, observational nature and other limitations. To elucidate this problem, a prospective study with a uniform reporting standard and careful design is required accordingly (23). Therefore, the utility of CL configuration requires further investigation, which propels us to launch this study.

This prospective, single-center, non-randomized controlled trial aims to compare the safety and efficacy of CL vs. SL configuration in EVAR patients, which could provide high-level evidence for choice of optimal stent graft configuration. The primary outcome of interest is 3-year freedom from major adverse limb-graft events (MALEs).

### Hypothesis to be tested

In AAA patients who receive EVAR, CL is not inferior to SL configuration in terms of safety and efficacy in the short and long term.

## Materials and methods

### Study design and approvals

This study is a prospective, single-center, non-randomized controlled study conducted in West China Hospital. This protocol is developed according to Standard Protocol Items: Recommendations for Interventional Trials (SPIRIT) 2013 Statement for study protocols of clinical trials (24). The SPIRIT checklist has been completed in additional file 1.

### Recruitment and enrollment

**FIGURE 1:**
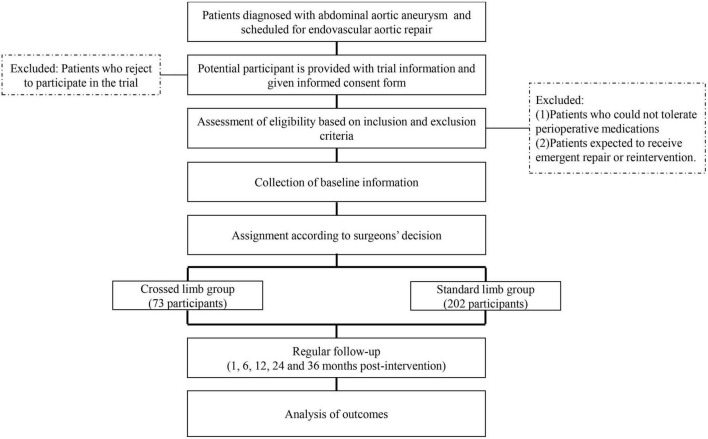
The procedure of screening, randomization and follow-up in the DANCER trial.

We recruit patients diagnosed with infrarenal AAA (Figure 1) from 1st December 2021. The eligibility criteria for inclusion and exclusion are shown in Table 1. Adult patients with indicated infrarenal AAA (diagnosed with ICD I71.3 and I71.4) and suitable iliofemoral anatomy are included in our study. Excluded are patients who could not tolerate perioperative medications and patients expected to receive reintervention. Both groups of patients share general inclusion or exclusion criteria.

**TABLE 1:** Inclusion and exclusion criteria for participants.

Inclusion	Exclusion
Patients are considered eligible if they meet all of the following criteria: 1. Men or non-pregnant women aged ≥ 18 years and with sufficient life expectancy to complete all the processes of the study. 2. Diagnosed with infrarenal abdominal aortic aneurysm (according to ICD I71.3 and I71.4) and indicated for surgical intervention. 3. Local anatomy of bilateral femoral and iliac arteries allows for guide wire passing. 4. Provide written informed consent and agree to participate in the study. 5. Placement of iliac endografts is required during the procedure.	Patients shall be excluded from the trial if they meet any of the following criteria: 1. Unable to tolerate contrast agents, antiplatelets or anticoagulants. 2. Recorded with previous primary repair for AAA, no matter EVAR or open surgical repair.

AAA, abdominal aortic aneurysm; EVAR, endovascular aneurysm repair.

**FIGURE 2:**
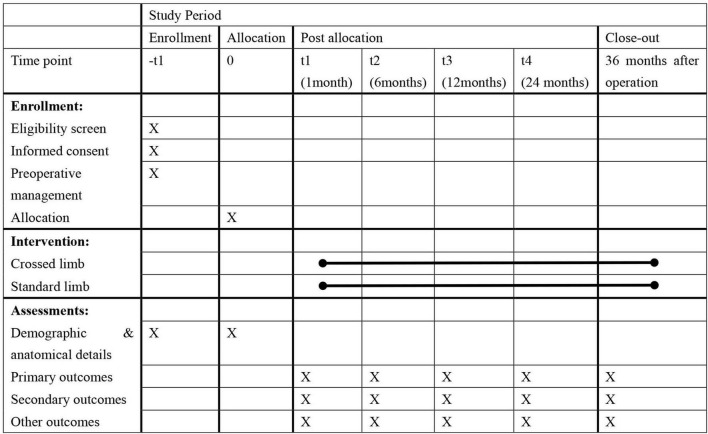
The schedule of enrollment, interventions and assessments.

We will recruit patients by putting up posters and advertising online to meet the expected enrollment. In addition, we provide fast-track follow-up for patients who are willing to participate in our study. Figure 2 shows the schedule of enrollment, interventions and assessments, following the template provided by SPIRIT (24).

### Allocations of intervention

Based on the anatomic features of the aneurysm by intraoperative imaging, surgeons will determine whether to adopt CL or SL configuration. When both configurations are feasible, surgeons will assign the limb configuration based on covariate adaptive matching between the two groups. The major matched covariates are as follows: age, gender and aneurysm diameter. Both surgeons and participants are not blinded to the interventions, and only statisticians who analyze the data are blinded.

The CL and SL configurations are defined as crossed or uncrossed limb grafts in anteroposterior view of the final angiogram, respectively. CL is further subdivided into anterior-posterior (AP) cross and left-right (LR) cross, which correspond to ≥ 50 and < 50% overlapped areas between two limb grafts in the angiogram. SL is similarly subdivided into AP parallel and LR parallel with the same definition.

The included patients will receive the same perioperative management, including blood pressure control, analgesia and medication with antiplatelets and statins. In interventional operating room, both common femoral arteries will be used for percutaneous or cut down access. After administration of 0.5 mg/kg unfractionated heparin, graft stents are placed in accordance with instruction for use by an experienced surgeon of vascular surgery. Treatment procedures are performed under a standard protocol. The Endurant II or IIs Stent Graft (Medtronic, Minneapolis, Minn) is used for repair.

Postoperative medications include antiplatelet monotherapy (aspirin 100 mg once a day or clopidogrel 75 mg once a day) and statins. Patients are expected to take these medications all life unless severe adverse event occurs. Anticoagulants, such as low molecular weight heparin or direct oral anticoagulants, will be used according to surgeons’ decision.

### Follow-up

After the procedure, all included patients will receive follow-up in outpatient clinics at 1, 6, and 12 months and annually thereafter. The required routine examinations include history taking, physical examination, blood biochemical index and duplex ultrasound. All biological specimens will be processed in routine procedure and not be used in ancillary or other studies. The computed tomography angiography will be performed to search for detailed information if any adverse event, especially endoleak or limb thrombosis, is found by duplex ultrasound. Patients who are unable to return to the outpatient clinic will be suggested to undergo duplex ultrasonography or computed tomography in local medical institutions, which will send medical records and images back to researchers. For patients who fail to finish the expected follow-up, telephone interview or WeChat message will be used instead to record survival status and postoperative adverse events. Due to no additional harm induced by the trial, all adverse events will be processed according to normal treatment procedure and patients will not automatically quit the group or receive additional compensation.

Patients are defined as dropout if the following events occur: (1) Fail to complete the expected follow-up and could not obtain image or medical records: a subject is considered lost to follow-up if they could not be contacted *via* five attempts by telephone or WeChat message. (2) Make withdrawal consent: all participants have the right to withdraw from the study at any time for any reason without obstruction.

### Outcomes of interest

The primary outcome is 3-year freedom from MALEs, which involves type IB/III endoleak and limb occlusion. Type IB endoleak is defined as persistent direct flow in the aneurysm sac due to inadequate distal seal of the stent graft. Type III endoleak is defined as persistent direct flow resulting from stent graft component separation or fabric tear. Limb occlusion is defined as a total occlusion occurring in limb grafts, regardless of symptoms, or an invasively treated stenosis resulting from thrombus formation (> 50% lumen reduction).

The secondary outcomes involve 3-year freedom from type IA endoleak and 3-year freedom from aortic reintervention. Type IA endoleak is defined as persistent direct flow in the aneurysm sac due to inadequate proximal seal of the stent graft. Aortic reintervention refers to any secondary surgical procedure related to the index EVAR.

Other outcomes consist of three aspects: intraoperative technical outcomes (operation time, radiation exposure time, dosage of contrast medium and technical success rate); short-term postoperative outcomes (length of stay in intensive care unit or vascular ward, acute kidney injury, 30-day overall morbidity, 30-day major adverse cardiac events, 30-day all-cause mortality); long-term outcomes (renal function decline, occurrence of type I/II/III endoleak or stent migration, freedom from major adverse cardiac events, aneurysm related death, overall survival).

Detailed information for outcomes is listed in Table 2.

**TABLE 2:** Detailed information for outcomes.

Outcomes	Definition
**Primary outcome**	
3-year freedom from MALEs	Free of type IB, III endoleak and limb occlusion within 3 years
**Secondary outcomes**	
3-year freedom from type IA endoleak	Free of type I endoleak within 3 years
3-year freedom from aortic reintervention	Free of any reintervention related to the index EVAR within 3 years
**Other outcomes**	
**Intraoperative technical outcome**	
technical success rate	Freedom from surgical conversion or mortality, type I or III endoleaks, or graft limb obstruction after the deployment of devices
**Short-term postoperative outcomes**	
Acute kidney injury	Serum creatinine elevates by 0.3 mg/dL or 50% compared with baseline within 48 h; or urine less than 0.5 mL/kg/h for more than 6 h
30-day morbidity	The existence of any adverse event within 30 days, consisting of infection, pseudoaneurysm, deep venous thrombosis, hypoalbuminemia, hemorrhage, embolism, stroke, buttock ischemia and systematic complications such as pulmonary, cardiac, cerebral and bowel events
30-day MACEs	Diagnosed with myocardial infarction, chronic cardiac failure, or receive repeat revascularization or died within 30 days
**Long-term outcomes**	
Renal function decline	Diagnosed with chronic renal failure and could not be attributed to other known causes
Type I endoleak	Endoleak due to inadequate proximal or distal seal of the stent graft
Type II endoleak	Endoleak originating from collateral vessels
Type III endoleak	Endoleak resulting from stent graft component separation or fabric tear
Stent migration	Migration from original position for more than 5 mm
Aneurysm related death	All deaths from secondary aneurysm rupture after repair, death within 30 days of any reintervention attributable to the aneurysm or death from other aneurysm-related causes (including graft infection or fistula)

MALEs, major adverse limb-graft events; EVAR, endovascular aneurysm repair; MACEs, major adverse cardiac events.

### Data collection

Figure 1 shows the flow diagram of the trial. Once patients are admitted, baseline data will be collected for both groups, including demographic characteristics, comorbidities and anatomical variables. The demographics are collected from history taking and hospital information system. The comorbidity is evaluated by the Charlson Comorbidity Index to quantify its severity. Anatomical variables are as follows: length of aneurysm neck, oversizing ratio in proximal neck and distal limb, neck angulation, maximum diameter of aneurysm sac, intramural thrombus load of aneurysmal sac, iliac tortuosity, common iliac artery aneurysm and distal iliac calcification. We will report reasons for withdrawal for each group and compare the reasons qualitatively. Details of the procedure, consisting of operation time, radiation exposure time and contrast medium dosage, are recorded immediately after the operations. At preoperative and postoperative evaluation (24, 48, and 72 h), laboratory examinations will be conducted and corresponding results will be collected from the laboratory information system.

### Quality control

The study staff have received systematic training for recording all baseline information before initiation of the project, with a special focus on recording comorbidity and measuring parameters of AAA. Pamphlets with protocols for history taking and definitions for related comorbidity have been distributed to the staff. By computed tomography angiography, all the diameters are measured from the minor axis of axial cuts or from planes perpendicular to the centerline in reformatted slices. Two researchers will independently complete the measurement and when more than 10% differences occur, a senior member will arbitrate and give final result. Medical staffs will strictly follow the standard procedure of AAA treatment in our hospital and provide the same perioperative management or nursing care for all patients.

### Sample size calculation

We calculate the sample size for this study based on the results of our previous study (22), in which 11.5 and 7.9% of patients suffered from adverse limb events in CL and SL groups, respectively. In this trial, we hypothesize that the freedom from adverse limb events in the CL group is not inferior to that in the SL group. The predetermined non-inferiority margin on the risk difference scale (δ) is set to 0.15 between the two groups. The type I and II error rates are set to 0.05 and 0.2, respectively. As the intraoperative choice of limb configuration is difficult to randomize, the ratio of enrolled patients between the CL and SL groups is set to 4:11 based on our previous cohort study (22). After accounting for a 20% rate of dropout, it is calculated that 73 subjects are needed for the CL group, and this value is 202 in the SL group. All calculations are performed by PASS 15 software.

### Statistical analysis

Categorical data is expressed as number and rate, while continuous data is expressed as means ± standard deviation if they are normally distributed or median with interquartile range otherwise. Student’s *t*-test or Mann-Whitney *U*-test is used for univariate analysis of continuous data. χ^2^-test or Fisher’s exact test is used for categorical data. For primary analyses, multivariate logistic regression is used to calculate adjusted odds ratio and 95% confidence interval for short-term outcomes. Cox proportional hazard regression analysis and marginal structural model are adopted to assess the association between limb configuration and time-to-event outcomes.

The propensity score to undergo CL or SL is estimated by logistic regression model based on demographic and anatomic information. Inverse probability of treatment weighting adjusted analyses are performed to achieve weighted balance with standardized differences < 0.10. Sensitivity analysis is conducted to stabilize the weights by truncating the non-overlapping tails of the propensity score distributions which below or above the 1st and 99th percentiles. Subgroup analysis is conducted in terms of large aneurysm sac, severely angulated neck, tortuous iliac arteries and iliac landing zone (whether using bell-bottom iliac stent graft). In addition, this analysis will be carried out in AP and LR subgroups. R studio Version 1.2.1335^1^ and Empower (X&Y solutions, Inc., Boston, MA)^2^ are utilized for statistical analysis.

### Patient and public involvement

Patients are also included in the design of the crosseD vs. stANdard Configuration in Endovascular Repair (DANCER). We conducted a preliminary survey in AAA patients who would receive EVAR to investigate the preferred follow-up modality and clinical outcomes they care most. Subsequent improvements were made accordingly.

## Discussion

**FIGURE 3:**
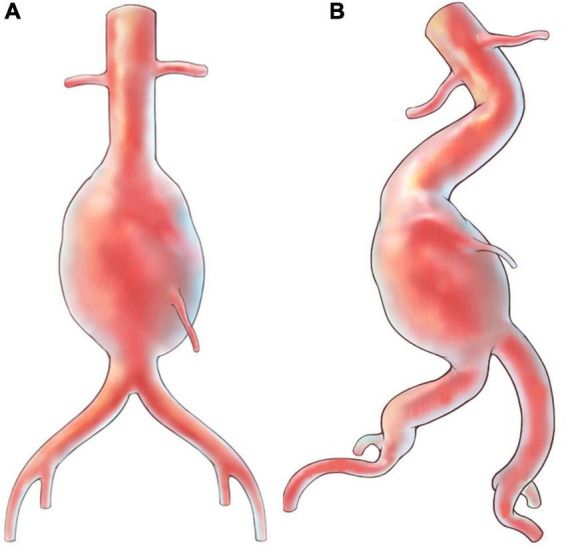
Illustrations for abdominal aortic aneurysm with hostile anatomy. **(A)** Aneurysm with “normal” appearance; **(B)** aneurysm with angulated neck and tortuous iliac arteries.

AAA is a localized dilation of infrarenal abdominal aorta. As a degenerative disease (25), it is common in the elderly population, with an estimated prevalence rate of 3.3% in men aged 65–74 years (26). AAA rupture is the most emergent case and has a high mortality rate even after immediate surgery (27). Therefore, prophylactic repair, either conducted by open surgery or EVAR, plays an important role in the treatment. EVAR has been recommended as a first-line modality for AAA (6, 7) and is widely applied in clinical practice. However, local vascular anatomy, in most cases referred to proximal neck, aneurysm sac, or aortoiliac arteries (Figure 3) (8), determines a successful EVAR performance, achieving sufficient sealing and fixation of anchoring segments (28). It could otherwise be technically challenging for the procedure, especially when cannulating contralateral gate of the endograft. Moreover, these anatomic predictors have a negative influence on postoperative outcomes. Angulated neck is found to be a predictor for sac enlargement (29), which is closely linked with postoperative aortic rupture (30). Sac remodeling, with a close connection with endoleaks, reintervention and mortality, may be affected by hostile neck and AAA volume (31). There is mounting evidence proving that patients with these hostile anatomical features could suffer from postoperative adverse events more commonly (32–35). As a result, for patients with hostile anatomy and serious comorbidities, clinicians may face a dilemma, as both EVAR and open surgery repair could be inapplicable.

**FIGURE 4:**
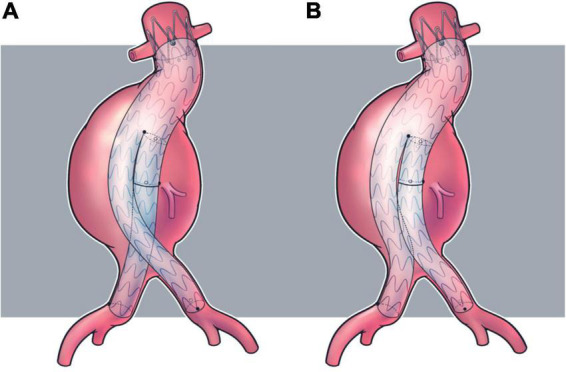
Illustrations for stent graft configuration. **(A)** Crossed limb; **(B)** standard limb.

Described by Ramaiah et al. in 2002 first (15), CL technique (Figure 4) is a useful adjunct to solve this problem by connecting ipsilateral guidewire to contralateral gate, arranging the limb graft just like a ballerina. It is expected to facilitate the procedure and, furthermore, reduce graft gate disconnection and endoleak. The true effect of CL, however, on postoperative outcomes has yet to be elucidated and currently no indications or contraindications have been described. This is a challenging issue for research because involved patients are always characterized by complicated anatomical conditions, which could induce considerable selection bias. Additionally, randomized controlled trials, with the highest evidence level, could be extremely difficult to conduct, as the performance of CL or SL is mainly based on anatomy and surgeons’ experience. Related studies, mainly cohort studies, have made inconsistent conclusions on this issue. Several studies revealed no difference in perioperative death, short or long term endoleak, limb graft occlusion, aneurysm sac expansion, reintervention or overall survival (19, 20), which was confirmed by a recent meta-analysis (21). While in our previous cohort study, which was characterized by the largest sample size and additional subgroup analysis, patients with large aneurysm sac and tortuous iliac arteries encountered fewer type I endoleaks in the CL group (22). This conclusion was consistent with a recent hemodynamic study revealing that this non-standard configuration could sustain higher wall shear stress and helicity characteristics (16, 17). However, it was questioned that it may be modified delivering system, instead of CL, that improved prognosis (36). Moreover, other computational analyses did not conclude likewise. Georgakarakos et al. revealed that the displacement force, one of the targets that CL aims at, was only slightly affected by the CL configuration and this effect could be blunted by concomitant modifications of the stent (37). Qing et al. studied the hemodynamic performance of these two configurations and concluded that the main factor affecting the index was the angle of aneurysm neck, while configuration had little effect on hemodynamics (18).

Several limitations exist in our study. Firstly, this is a non-randomized controlled trial and therefore it could not provide higher level of evidence like randomized one. However, as discussed above, we believe that this is the most suitable research type for this issue and could draw convincing conclusions similarly. Secondly, we conduct this trial in a single center in West China Hospital, which is a tertiary medical center and receive many patients with complex medical problems. This may cause heterogeneity and whether this conclusion could be generalized to the world is uncertain. The large sample number we anticipated, however, could make up for this problem partly.

Currently, the true effect of CL on clinical outcomes is still a matter of debate. The lack of a higher level of evidence, especially prospective clinical trials in this scenario, prevents its elucidation. Therefore, the main aim of our present trial is to compare the safety and efficacy between CL and SL configuration in patients receiving EVAR with Endurant II or IIs Stent Graft. Moreover, two spatial subclassifications of CL and SL, AP and LR, will be further investigated. This will hopefully provide high-level evidence for limb graft placement in AAA patients, especially in those with hostile anatomy.

## Ethics statement

The trial was approved by the Ethics Committee on Biomedical Research, West China Hospital of Sichuan University (approval number: 202–1353) on 8th November 2021 and registered in the Chinese Clinical Trial Registry (registration number: ChiCTR2100053055) on 10th November 2021. The studies involving human participants were reviewed and approved by Ethics Committee on Biomedical Research, West China Hospital of Sichuan University. The patients/participants will all provide their written informed consent to participate in this study.

## Author contributions

JZ was the project leader. YS and JW wrote the manuscript of the protocol. JW was responsible for calculating the sample size and performing data analysis. BH and TW contributed to devise the study concept. All authors contributed to patient recruitment, data collection and EVAR performance, read, and approved the final manuscript.

## Footnotes

1
http://www.R-project.org

2
www.empowerstats.com

## Abbreviations

AAA: abdominal aortic aneurysm
AP: anterior-posterior
CL: crossed limb
DANCER: crosseD vs. stANdard Configuration in Endovascular Repair
EVAR: endovascular aneurysm repair
LR: left-right
MALEs: major adverse limb-graft events
SL: standard limb
SPIRIT: Standard Protocol Items: Recommendations for Interventional Trials.
